# Development of novel DNA marker for species discrimination of *Fasciola* flukes based on the fatty acid binding protein type I gene

**DOI:** 10.1186/s13071-022-05538-7

**Published:** 2022-10-20

**Authors:** Emi Okamoto, Michiyo Tashiro, Pedro Ortiz, Uday Kumar Mohanta, Cristian Hobán, César A. Murga-Moreno, José M. Angulo-Tisoc, Madoka Ichikawa-Seki

**Affiliations:** 1 Iwate University, Morioka, Japan; 2 Universidad Nacional de Cajamarca, Cajamarca, Peru; 3 Sher-E-Bangla Agricultural University, Dhaka, Bangladesh; 4 Universidad Nacional Mayor de San Marcos, Cusco, Peru

**Keywords:** *Fasciola*, Multiplex PCR, Genotyping, *FABP* type I

## Abstract

**Background:**

Multiplex polymerase chain reaction (PCR) and PCR-restriction fragment length polymorphism (RFLP) for nuclear phosphoenolpyruvate carboxykinase (*pepck*) and polymerase delta (*pold*), respectively, have been used to differentiate *Fasciola hepatica*, *F. gigantica*, and hybrid *Fasciola* flukes. However, discrimination errors have been reported in both methods. This study aimed to develop a multiplex PCR based on a novel nuclear marker, the fatty acid binding protein type I (*FABP*) type I gene.

**Methods:**

Nucleotide sequence variations of FABP type I were analyzed using DNA samples of *F. hepatica*, *F. gigantica*, and hybrid *Fasciola* flukes obtained from 11 countries in Europe, Latin America, Africa, and Asia. A common forward primer for *F. hepatica* and *F. gigantica* and two specific reverse primers for *F. hepatica* and *F. gigantica* were designed for multiplex PCR.

**Results:**

Specific fragments of *F. hepatica* (290 bp) and *F. gigantica* (190 bp) were successfully amplified using multiplex PCR. However, the hybrid flukes contained fragments of both species. The multiplex PCR for FABP type I could precisely discriminate the 1312 *Fasciola* samples used in this study. Notably, no discrimination errors were observed with this novel method.

**Conclusions:**

Multiplex PCR for *FABP* type I can be used as a species discrimination marker in place of *pepck* and *pold*. The robustness of the species-specific primer should be continuously examined using a larger number of *Fasciola* flukes worldwide in the future since nucleotide substitutions in the primer regions may cause amplification errors.

**Graphical abstract:**

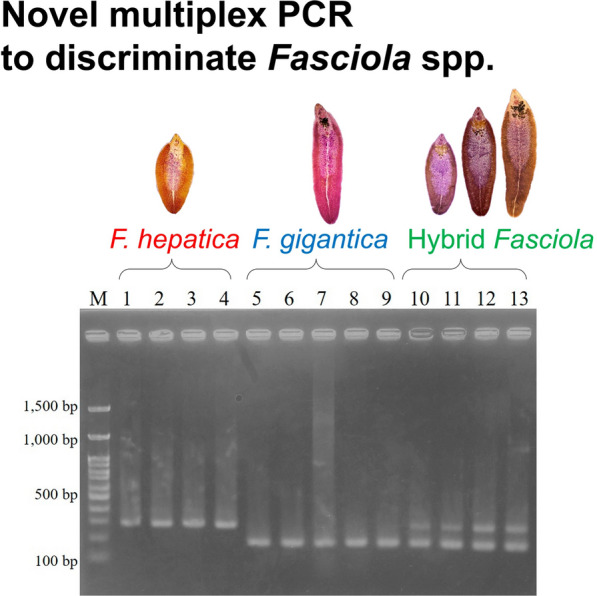

**Supplementary Information:**

The online version contains supplementary material available at 10.1186/s13071-022-05538-7.

## Background

Fasciolosis causes huge economic losses to the livestock industry in endemic areas [1, 2]. *Fasciola hepatica* and *F. gigantica* are well-known causative agents of this disease. Both species have normal spermatogenic abilities and reproduce bisexually by fertilization. In contrast, the hybrid *Fasciola* flukes of the two species have been reported in many Asian countries [3]. Both diploids and triploids have been reported in hybrid *Fasciola* flukes [4, 5]. Because hybrid flukes harbor a meiotic disorder that affects spermatogenesis, they probably reproduce parthenogenetically [5]. Therefore, it is important to precisely discriminate hybrid flukes from *F. hepatica* and *F. gigantica* because they are speculated to have stronger viability than the two species [6].

Multiplex polymerase chain reaction (PCR) and PCR restriction fragment length polymorphism (RFLP) for nuclear phosphoenolpyruvate carboxykinase (*pepck*) and polymerase delta (*pold*), respectively, can differentiate *Fasciola* spp. by the fragment patterns of *F. hepatica* (Fh), *F. gigantica* (Fg), and the hybrid (both Fh and Fg: Fh/Fg) [7]. The existence of the Fh/Fg type in the two nuclear markers suggests that hybrid *Fasciola* flukes are descendants originating from the hybridization of *F. hepatica* and *F. gigantica* [3, 6].

Although discrimination errors in the fragment pattern analysis of the multiplex PCR for *pepck* have been reported in *F. hepatica* isolates from Afghanistan [8], Algeria [9], Ecuador [10], and Spain [11], subsequent nucleotide sequencing of DNA fragment of *pepck* enabled precise species identification. Regarding *pold*, discrimination errors were observed in *F. gigantica* isolates from Nigeria [12]. A single-nucleotide substitution at the recognition site of the restriction enzyme was identified as the cause of the error in PCR-RFLP [12].

Fatty acid binding protein (*FABP*) type I of *Fasciola* flukes encoded in the nuclear DNA has multifunctional roles, such as immune modulation and anthelmintic sequestration [13]. Moreover, the messenger RNA (mRNA) sequence of *FABP* type I is available in the DNA databank [13]. This study analyzed the nucleotide sequence variations of *FABP* type I in *F. hepatica*, *F. gigantica*, and hybrid *Fasciola* flukes. Then, a multiplex PCR for *FABP* type I was developed and applied to 1312 *Fasciola* spp. from 11 countries in Asia, Africa, Europe, the Near and Middle East, and Latin America. The novel multiplex PCR for *FABP* type I was proven to be a useful marker in place of *pepck* and *pold* for precise species discrimination of *Fasciola* spp.

## Methods

### *Fasciola* samples

A total of 1312 *Fasciola* flukes (470 *F. hepatica*, 609 *F. gigantica*, and 233 hybrid *Fasciola*) from 11 countries (Afghanistan, Algeria, Peru, Spain, Indonesia, Malaysia, Nigeria, Pakistan, Uganda, Japan, and Bangladesh) [8, 9, 11, 12, 14–20] were used in the present study. Fragment analyses of nuclear *pepck* and *pold* and the nucleotide sequencing of mitochondrial *nad1* have been performed in previous studies [8, 9, 11, 12, 14–20]. Discrepancies between *pepck* and *pold* were observed among 7, 19, 6, 27, and 15 *Fasciola* isolates from Afghanistan, Algeria, Peru, Spain, and Nigeria, respectively. All available information on the *Fasciola* samples is summarized in Table 1.

**Table 1:** Nuclear marker profiles of *Fasciola* flukes used in this study

Species	Country	Sample number	*FABP* type I (multiplex PCR)			*pepck* (multiplex PCR)			*pold* (PCR–RFLP)		
			Fh	Fg	Fh/Fg	Fh	Fg	Fh/Fg	Fh	Fg	Fh/Fg
*F. hepatica*	Afghanistan [8]	92	92	0	0	85	0	7	92	0	0
	Algeria [9]	68	68	0	0	49	1	18	68	0	0
	Peru [14] ^a^	114	114	0	0	108	1	5	114	0	0
	Spain [11]	196	196	0	0	169	1	26	196	0	0
Subtotal		470	470	0	0	411	3	56	470	0	0
*F. gigantica*	Indonesia [15]	60	0	60	0	0	60	0	0	60	0
	Malaysia [20]	36	0	36	0	0	36	0	0	36	0
	Nigeria [12]	172	0	172	0	0	172	0	0	157	15
	Pakistan [17]	49	0	49	0	0	49	0	0	49	0
	Uganda [18]	292	0	292	0	0	292	0	0	292	0
Subtotal		609	0	609	0	0	609	0	0	594	15
Hybrid	Japan [16] ^b^	201	0	0	201	0	0	201	0	0	201
	Bangladesh ^c^	32	0	0	32	0	0	32	0	0	32
Subtotal		233	0	0	233	0	0	233	0	0	233
Total		1312	470	609	233	411	612	289	470	594	248

^a^Seventy-eight *Fasciola* flukes were used in the previous study. The remaining samples were analyzed in the present study

^b^The results of *pold* were obtained in the present study

^c^Analyzed in the present study

Some of the analyses for *pepck* and *pold* were conducted in the present study. Briefly, a small portion of the vitelline glands from the posterior part of each fluke was used for DNA extraction using the High Pure PCR Template Preparation Kit (Roche, Mannheim, Germany), following the manufacturer’s protocols, and stored at – 20 °C until further use. Fragments of *pepck* were amplified using a multiplex PCR assay with Fh-pepck-F (5′-GATTGCACCGTTAGGTTAGC-3′), Fg-pepck-F (5′-AAAGTTTCTATCCCGAACGAAG-3′), and Fcmn-pepck-R (5′-CGAAAATTATGGCATCAATGGG-3′) primers based on a previous study [7]. PCR amplicons were electrophoresed on 1.8% agarose gels for 30 min to detect fragment patterns for *F. hepatica* (approximately 500 bp), *F. gigantica* (approximately 240 bp), or hybrid (both fragments). The fragments of *pold* were analyzed using the PCR-RFLP assay described in a previous study [7]. The PCR products were amplified using Fasciola-pold-F1 (5′-GCTAACTTATCTGCTTACACGTGGACA-3′) and Fasciola-pold-R1 (5′-ATCGCATTCGATCAAAGCCCTCCCATG-3′) and subsequently digested with *Alu*I enzyme (Toyobo, Osaka, Japan) at 37 °C for 3 h. The resulting products were electrophoresed on 1.8% agarose gels for 30 min to detect fragment patterns for *F. hepatica* (approximately 700 bp), *F. gigantica* (approximately 500 bp), or hybrid (both fragments).

### Sequence determination of *FABP* type I

A primer set, FABP type I-F(5′-CACGATGGCTGACTTTGTGG-3′) and FABP type I-R(5′-AATTTTATTTGTCAGTGTTGTCGG-3′), was designed based on the mRNA sequence of *FABP* type I generated from *F. hepatica* (accession no. M95291) [13].

PCRs were performed for *F. hepatica* isolates from Peru, and *F. gigantica* isolates from Uganda in a 25 μl reaction mixture containing 2 µl template DNA, 0.2 μM of each primer, 1 U of Gflex polymerase (Takara Bio, Shiga, Japan), and the manufacturer’s supplied reaction buffer. Thermal conditions included an initial denaturation step at 94 °C for 60 s, followed by 30 cycles of 98 °C for 10 s, 60 °C for 15 s, and 68 °C for 180 s. Fragments of approximately 3000 bp were amplified and purified using the NucleoSpin Gel and PCR Clean-up kit (MACHEREY–NAGEL, Düren, Germany) and then directly sequenced from both directions to obtain the preliminary sequences of *FABP* type I. An inner primer set, FABP type I-2F (5′-CTGGTGATGTTGAGAAGG-3′) and FABP type I-2R(5′-ACTCGTCGTCGTTTACACCCTC-3′), was generated to amplify *partial FABP type I* gene in *F. hepatica* (1951 bp) and *F. gigantica* (1961 bp), respectively. PCR conditions were almost the same as that described above, except for the annealing temperature, 55 °C. The nucleotide sequences of the PCR amplicons were determined precisely.

**Fig. 1:**
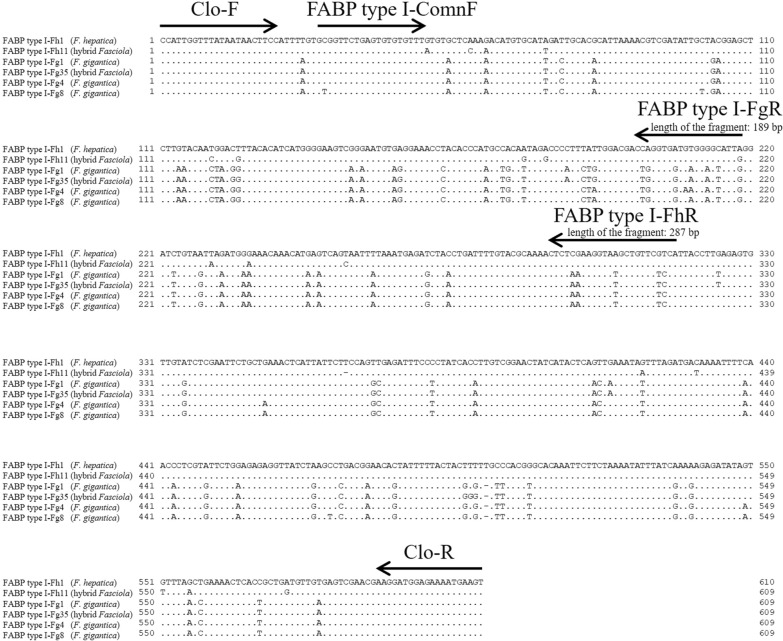
Alignment of partial *FABP* type I gene to generate primers. Six representative genotypes of *FABP* type I were analyzed using Clo-F and Clo-R. A dot in the alignment indicates that the sequence was identical to that of *FABP* type I-Fh1 (*Fasciola hepatica*). The arrows indicate the position and direction of the primers. The horizontal bars represent the alignment gap. Three primers (FABP type I-ComnF, FABP type I-FhR, and FABP type I-FgR) were designed for the multiplex PCR

Another inner primer set, Clo-F (5′-CCATTGGTTTATAATAACTTCC-3′) and Clo-R (5′-ACTTCATTTTCTCCATCCTT-3′), which could amplify an intron of *FABP* type I, was designed to examine nucleotide variations between the primer regions (*F. hepatica*: 567 or 568 bp; *F. gigantica*: 566 or 567 bp) (Fig. 1). Sequence determination between Clo-F and Clo-R was performed for approximately 5% of *F. hepatica* and *F. gigantica* as well as 10 hybrid flukes selected from each country, and flukes with different *nad1* haplotypes were selected as much as possible to ensure variations in the samples (Additional file 1: Table S1). PCRs were performed in a 25 μl reaction mixture containing 2 μl template DNA, 0.4 mM of each dNTP, 0.3 μM of each primer (Clo-F and Clo-R), 1 U of KOD FX Neo (Toyobo, Osaka, Japan), and the manufacturer’s supplied reaction buffer. Thermal conditions included an initial denaturation step at 94 °C for 120 s, followed by 35 cycles of 98 °C for 10 s, 50 °C for 30 s, and 68 °C for 30 s. PCR products were purified using the NucleoSpin Gel and PCR Clean-up kit (Macherey-Nagel), cloned into the pUC118 *Hinc II/BAP* vector (Takara Bio), and sequenced. Two clones were analyzed for *F. hepatica* and *F. gigantica*, whereas four clones (two for *F. hepatica* genotype and two for *F. gigantica* genotype) were analyzed for the hybrid *Fasciola* fluke (Additional file 1: Table S1). The obtained sequences were aligned to construct a maximum likelihood (ML) tree using MEGA 10.0.5 software [21]. For ML tree construction, all sites were selected in the gaps/missing data treatment, and the T92 + I model was used.

### Multiplex PCR

A primer set for multiplex PCR was designed using the resulting sequences of Clo-F and Clo-R. FABP type I-ComnF (5-′GCGGTTCTGAGTGTGTGTTT-3′) is a common primer for *F. hepatica* and *F. gigantica*, whereas FABP type I-FhR (5′-TGACGAACAGCTTACCTTCGAG-3′) and FABP type I-FgR (5′-CAATACTCCTCACCACCCAG-3′) are specific to *F. hepatica* (length of the amplicon: 287 bp) and *F. gigantica* (189 bp), respectively (Fig. 1). PCR amplification was performed in 10 μl reaction mixtures containing 0.5 µl template DNA, 0.1 µM of each dNTP, 0.2 µM of each primer, 0.01 U of Go *Taq* DNA Polymerase (Promega, Madison, WI, USA), and the manufacturer’s supplied reaction buffer. The PCR conditions included an initial denaturation step at 95 °C for 120 s, followed by 35 cycles of 95 °C for 30 s, 55 °C for 30 s, and 72 °C for 60 s, and a final extension step at 72 °C for 5 min. PCR amplicons were electrophoresed on 1.8% agarose gels and visualized using ethidium bromide staining. Multiplex PCR was then applied to all 1312 flukes (Table 1).

## Results and discussion

**Fig. 2:**
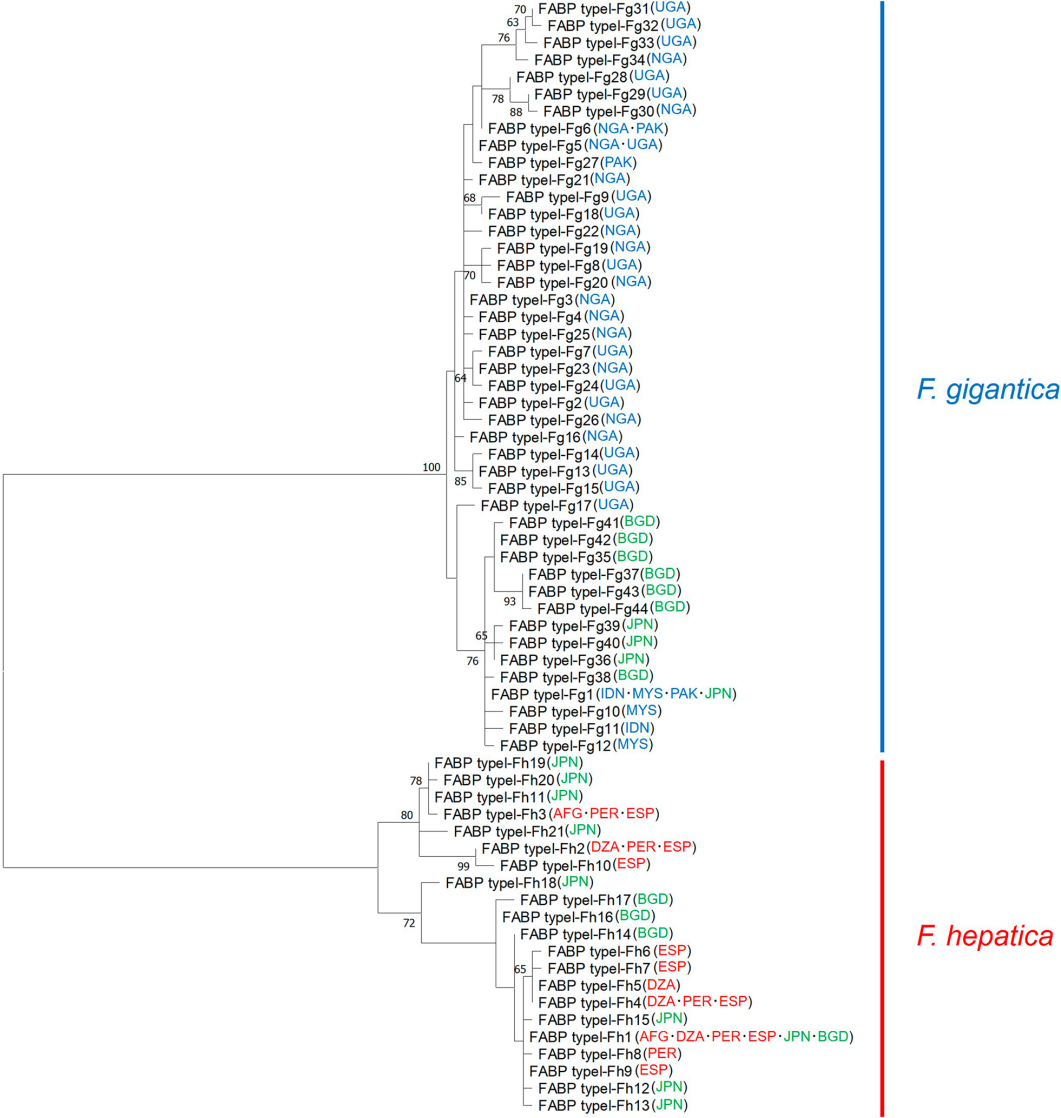
Maximum-likelihood tree of *FABP* type I genotypes. Sequences obtained with Clo-F and Clo-R primers were used in the tree. Bootstrap values > 60% are shown for the tree node. No suitable outgroups were available in the DNA databank. Abbreviations of the names of countries where each genotype was detected are mentioned on the tree. AFG: Afghanistan; DZA: Algeria; PER: Peru; ESP: Spain; IDN: Indonesia; MYS: Malaysia; NGA: Nigeria; PAK: Pakistan; UGA: Uganda; JPN: Japan; BGD: Bangladesh. Red, blue, and green indicate the regions where *F. hepatica*, *F. gigantica*, and hybrid *Fasciola* used in the present study were collected

The nucleotide sequences of PCR amplicons generated by FABP type I-2F and R for *F. hepatica* (1951 bp) and *F. gigantica* (1961 bp) were deposited in the DNA data bank of Japan (DDBJ) under accession numbers LC718926 and LC718927, respectively. The shorter nucleotide sequences of 24 *F. hepatica*, 31 *F. gigantica*, and 20 hybrid *Fasciola* flukes amplified using Clo-F and Clo-R were determined by cloning analysis (Additional file 1: Table S1). As a result, 10 genotypes (FABP type I-Fh1 to Fh10) were detected from *F. hepatica*, and 34 genotypes (FABP type I-Fg1 to Fg34) were detected from *F. gigantica*. Moreover, 12 *F. hepatica* (FABP type I-Fh1 and from FABP type I-Fh11 to Fh21) and 11 *F. gigantica* genotypes (FABP type I-Fg1 and from FABP type I-Fg35 to Fg44) were found in the hybrid *Fasciola* flukes. They were deposited in the DDBJ under accession numbers LC718928–LC718992 (Additional file 1: Table S1). FABP type I-Fh1 was detected in both *F. hepatica* and the hybrid *Fasciola* (Additional file 1: Table S1). FABP type I-Fg1 was found in both *F. gigantica* and the hybrid flukes (Additional file 1: Table S1). These observations may indicate an ancestor-descendant relationship. However, geographical distribution of the *FABP* type I genotypes among the 11 countries was not clear (Fig. 2).

**Fig. 3:**
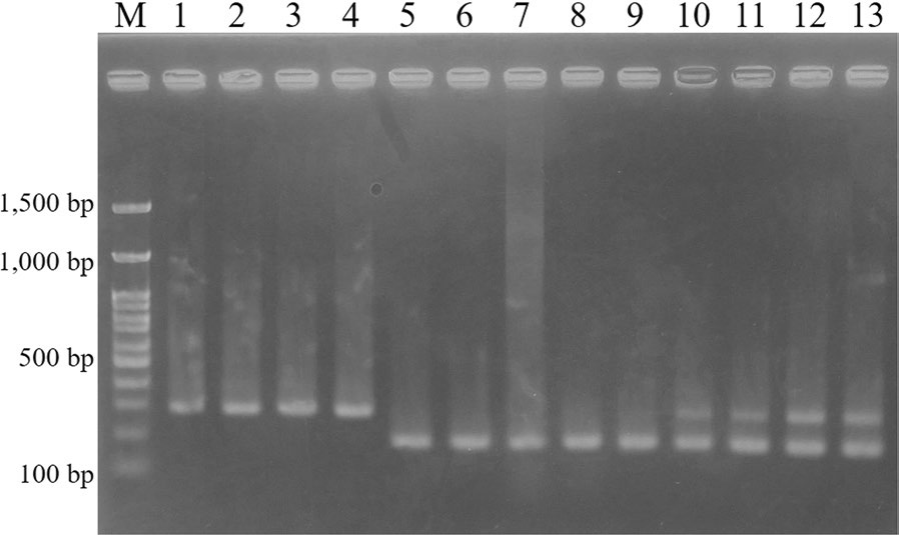
Multiplex PCR for *FABP* type I *Fasciola hepatica* (Fh type) from 1. Afghanistan, 2. Algeria, 3. Peru, and 4. Spain. *F. gigantica* (Fg type) from 5. Indonesia, 6. Malaysia, 7. Nigeria, 8. Pakistan, and 9. Uganda. Hybrid *Fasciola* flukes (Fh/Fg type) from 10, 11. Japan and 12, 13. Bangladesh. M: 100-bp DNA ladder

The *FABP* type I genotypes obtained in this study were clearly divided into the clades of *F. hepatica* and *F. gigantica* (Fig. 2). Therefore, the nucleotide variations of *FABP* type I are sufficient to distinguish *F. hepatica* and *F. gigantica* genotypes and therefore can be regarded as a useful molecular discrimination marker. The species-specific primers developed for multiplex PCR successfully generated specific fragments of *F. hepatica* (approximately 290 bp) and *F. gigantica* (approximately 190 bp), whereas hybrid flukes had both fragment patterns (Fig. 1 and 3).

**Fig. 4:**
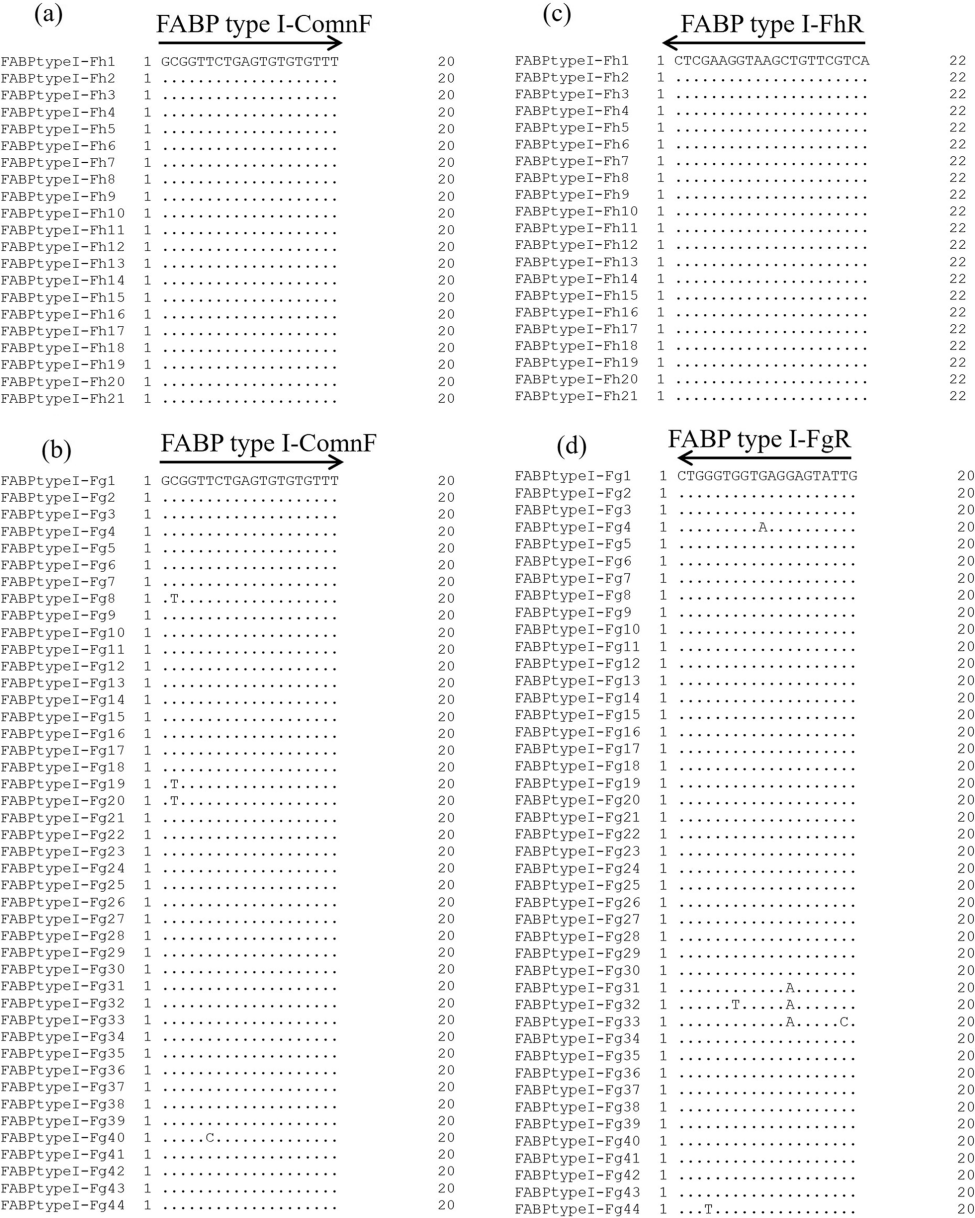
Nucleotide variations in the primer region of the multiplex PCR. **a** Nucleotide variations of FABP type I-ComnF region. *Fasciola hepatica* genotypes. **b** Nucleotide variations of FABP type I-ComnF region. *F. gigantica* genotypes. **c** Nucleotide variations of FABP type I-FhR. **d** Nucleotide variations of FABP type I-FgR

No mutation was found in the FABP type I-ComnF primer region of *F. hepatica*, whereas a single-nucleotide mutation was found in the four *F. gigantica* genotypes (Fig. 4a and b). Similarly, no mutation was detected in the FABP type I-FhR region, but one to two nucleotide substitutions were observed in five *F. gigantica* genotypes in the FABP type I-FgR region (Fig. 4c and d). However, these mutations did not interfere with the DNA amplification of the multiplex PCR in this study because no ambiguous or variant fragments were detected (Table 1).

Previous studies observed discrepancies in 7, 19, 6, and 27 *F. hepatica* isolates from Afghanistan, Algeria, Peru, and Spain, respectively. They displayed the Fg or Fh/Fg type in the *pepck* (Table 1). However, in this study, all of them displayed Fh fragment patterns in the multiplex PCR for *FABP* type I, and there was no discrepancy when compared with the results of *pold* (Table 1). Moreover, the 15 *F. gigantica* from Nigeria showed Fg type in the multiplex PCR for *FABP* type I, which coincided with the results of *pepck*, even though they displayed an Fh/Fg-like fragment pattern in the *pold* (Table 1). Therefore, the novel multiplex PCR for *FABP* type I proved to be a useful marker to replace *pepck* and *pold*.

## Conclusions

We successfully developed a novel multiplex PCR based on *FABP* type I using 1312 *Fasciola* flukes from 11 countries. Although discrimination errors occurring in *pepck* and *pold* were completely resolved by fragment analysis of *FABP* type I (Table 1), the robustness of the species-specific primer should be examined continuously in the future using a larger number of *Fasciola* flukes worldwide as nucleotide variations were detected in the primer regions.

## Supplementary Information

**Additional file 1: Table S1.** Fatty acid binding protein type I (*FABP* type I) genotypes of *Fasciola* flukes used in the present study.

## Data Availability

The nucleotide sequences obtained in this study are available under accession nos. LC718926 to LC718992.

## Abbreviations

*FABP* type I: Fatty acid binding protein type I
PCR: Polymerase chain reaction
PCR-RFLP: PCR-restriction fragment length polymorphism
*pepck*: Nuclear phosphoenolpyruvate carboxykinase
*pold*: Polymerase delta
mRNA: Messenger RNA
