# Supplementary material for: Development of novel DNA marker for species discrimination of Fasciola flukes based on the fatty acid binding protein type I gene
Source: Parasit Vectors. 2022 Oct 20;15:379. doi: 10.1186/s13071-022-05538-7 (PMC9585863; doi:10.1186/s13071-022-05538-7)
Supplement: Supplementary file 1 — Additional file 1: Table S1. Fatty acid binding protein type I (FABP type I) genotypes of Fasciola flukes used in the present study. [file 13071_2022_5538_MOESM1_ESM.docx]

Table S1 Fatty acid binding protein type I (*FABP type I*) genotypes of *Fasciola* flukes used in the present study.

| Species | Country | No. of flukes | *FABP type I* genotype | No. of clones | *pepck* | *pold* | *nad1* |
| --- | --- | --- | --- | --- | --- | --- | --- |
| *F. hepatica* | Afghanistan | 5 | FABP type I-Fh1 | 2 | Fh | Fh | AFG-nad1Fh1 |
|  |  |  | FABP type I-Fh1 | 2 | Fh/Fg | Fh | AFG-nad1Fh2 |
|  |  |  | FABP type I-Fh1 | 1 | Fh/Fg | Fh | AFG-nad1Fh10 |
|  |  |  | FABP type I-Fh3 | 1 |  |  |  |
|  |  |  | FABP type I-Fh1 | 2 | Fh | Fh | AFG-nad1Fh11 |
|  |  |  | FABP type I-Fh1 | 2 | Fh/Fg | Fh | AFG-nad1Fh19 |
|  | Algeria | 3 | FABP type I-Fh2 | 2 | Fh/Fg | Fh | DZ-nad1Fh1 |
|  |  |  | FABP type I-Fh1 | 2 | Fh/Fg | Fh | DZ-nad1Fh1 |
|  |  |  | FABP type I-Fh4 | 1 | Fh/Fg | Fh | DZ-nad1Fh22 |
|  |  |  | FABP type I-Fh5 | 1 |  |  |  |
|  | Peru | 6 | FABP type I-Fh1 | 1 | Fh | Fh | ND1-P1 |
|  |  |  | FABP type I-Fh2 | 1 |  |  |  |
|  |  |  | FABP type I-Fh1 | 2 | Fh | Fh | ND1-P7 |
|  |  |  | FABP type I-Fh1 | 2 | Fh | Fh | ND1-P2 |
|  |  |  | FABP type I-Fh1 | 1 | Fh | Fh | ND1-P3 |
|  |  |  | FABP type I-Fh4 | 1 |  |  |  |
|  |  |  | FABP type I-Fh3 | 1 | Fh | Fh | ND1-P6 |
|  |  |  | FABP type I-Fh8 | 1 |  |  |  |
|  |  |  | FABP type I-Fh4 | 1 | Fh/Fg | Fh | ND1-P8 |
|  |  |  | FABP type I-Fh3 | 1 |  |  |  |
|  | Spain | 10 | FABP type I-Fh1 | 1 | Fh | Fh | ES-Fh1 |
|  |  |  | FABP type I-Fh9 | 1 |  |  |  |
|  |  |  | FABP type I-Fh1 | 1 | Fh | Fh | ES-Fh26 |
|  |  |  | FABP type I-Fh2 | 1 |  |  |  |
|  |  |  | FABP type I-Fh1 | 2 | Fh/Fg | Fh | ES-Fh1 |
|  |  |  | FABP type I-Fh3 | 1 | Fh | Fh | ES-Fh14 |
|  |  |  | FABP type I-Fh7 | 1 |  |  |  |
|  |  |  | FABP type I-Fh1 | 2 | Fh/Fg | Fh | ES-Fh9 |
|  |  |  | FABP type I-Fh2 | 2 | Fh/Fg | Fh | ES-Fh2 |
|  |  |  | FABP type I-Fh1 | 2 | Fh | Fh | ES-Fh5 |
|  |  |  | FABP type I-Fh1 | 1 | Fh | Fh | ES-Fh21 |
|  |  |  | FABP type I-Fh10 | 1 |  |  |  |
|  |  |  | FABP type I-Fh1 | 1 | Fh | Fh | ES-Fh22 |
|  |  |  | FABP type I-Fh4 | 1 |  |  |  |
|  |  |  | FABP type I-Fh1 | 1 | Fh/Fg | Fh | ES-Fh11 |
|  |  |  | FABP type I-Fh6 | 1 |  |  |  |
| *F. gigantica* | Indonesia | 3 | FABP type I-Fg1 | 2 | Fg | Fg | ND1-ID-1 |
|  |  |  | FABP type I-Fg1 | 2 | Fg | Fg | ND1-ID-19 |
|  |  |  | FABP type I-Fg1 | 1 | Fg | Fg | ND1-ID-12 |
|  |  |  | FABP type I-Fg11 | 1 |  |  |  |
|  | Malaysia | 2 | FABP type I-Fg1 | 1 | Fg | Fg | MY-1(ID-1) |
|  |  |  | FABP type I-Fg12 | 1 |  |  |  |
|  |  |  | FABP type I-Fg1 | 1 | Fg | Fg | MY-3(T7) |
|  |  |  | FABP type I-Fg10 | 1 |  |  |  |
|  | Nigeria | 9 | FABP type I-Fg6 | 1 | Fg | Fg | NG42 |
|  |  |  | FABP type I-Fg26 | 1 |  |  |  |
|  |  |  | FABP type I-Fg3 | 1 | Fg | Fg | NG58 |
|  |  |  | FABP type I-Fg25 | 1 |  |  |  |
|  |  |  | FABP type I-Fg3 | 2 | Fg | Fg | NG1 |
|  |  |  | FABP type I-Fg5 | 1 | Fg | Fg | NG4 |
|  |  |  | FABP type I-Fg34 | 1 |  |  |  |
|  |  |  | FABP type I-Fg4 | 2 | Fg | Fg | NG6 |
|  |  |  | FABP type I-Fg4 | 1 | Fg | Fg | NG21 |
|  |  |  | FABP type I-Fg30 | 1 |  |  |  |
|  |  |  | FABP type I-Fg19 | 1 | Fg | Fg | NG5 |
|  |  |  | FABP type I-Fg21 | 1 |  |  |  |
|  |  |  | FABP type I-Fg20 | 1 | Fg | Fg | NG2 |
|  |  |  | FABP type I-Fg22 | 1 |  |  |  |
|  |  |  | FABP type I-Fg16 | 1 | Fg | Fg | NG2 |
|  |  |  | FABP type I-Fg23 | 1 |  |  |  |
|  | Pakistan | 2 | FABP type I-Fg1 | 2 | Fg | Fg | PAK-Fg9 |
|  |  |  | FABP type I-Fg6 | 1 | Fg | Fg | PAK-Fg1 |
|  |  |  | FABP type I-Fg27 | 1 |  |  |  |
|  | Uganda | 15 | FABP type I-Fg2 | 1 | Fg | Fg | UG-nad1Fg1 |
|  |  |  | FABP type I-Fg32 | 1 |  |  |  |
|  |  |  | FABP type I-Fg9 | 1 | Fg | Fg | UG-nad1Fg2 |
|  |  |  | FABP type I-Fg29 | 1 |  |  |  |
|  |  |  | FABP type I-Fg9 | 1 | Fg | Fg | UG-nad1Fg3 |
|  |  |  | FABP type I-Fg18 | 1 |  |  |  |
|  |  |  | FABP type I-Fg2 | 1 | Fg | Fg | UG-nad1Fg4 |
|  |  |  | FABP type I-Fg31 | 1 |  |  |  |
|  |  |  | FABP type I-Fg2 | 1 | Fg | Fg | UG-nad1Fg5 |
|  |  |  | FABP type I-Fg14 | 1 |  |  |  |
|  |  |  | FABP type I-Fg2 | 2 | Fg | Fg | UG-nad1Fg6 |
|  |  |  | FABP type I-Fg2 | 2 | Fg | Fg | UG-nad1Fg7 |
|  |  |  | FABP type I-Fg7 | 2 | Fg | Fg | UG-nad1Fg8 |
|  |  |  | FABP type I-Fg2 | 2 | Fg | Fg | UG-nad1Fg9 |
|  |  |  | FABP type I-Fg8 | 2 | Fg | Fg | UG-nad1Fg52 |
|  |  |  | FABP type I-Fg5 | 1 | Fg | Fg | UG-nad1Fg24 |
|  |  |  | FABP type I-Fg33 | 1 |  |  |  |
|  |  |  | FABP type I-Fg13 | 1 | Fg | Fg | UG-nad1Fg101 |
|  |  |  | FABP type I-Fg15 | 1 |  |  |  |
|  |  |  | FABP type I-Fg2 | 1 | Fg | Fg | UG-nad1Fg63 |
|  |  |  | FABP type I-Fg24 | 1 |  |  |  |
|  |  |  | FABP type I-Fg2 | 1 | Fg | Fg | UG-nad1Fg74 |
|  |  |  | FABP type I-Fg17 | 1 |  |  |  |
|  |  |  | FABP type I-Fg2 | 1 | Fg | Fg | UG-nad1Fg29 |
|  |  |  | FABP type I-Fg28 | 1 |  |  |  |
| Hybrid | Japan | 10 | FABP type I-Fh1 | 1 | Fh/Fg | Fh/Fg | Fsp1 |
|  |  |  | FABP type I-Fh21 | 1 |  |  |  |
|  |  |  | FABP type I-Fg1 | 1 |  |  |  |
|  |  |  | FABP type I-Fg36 | 1 |  |  |  |
|  |  |  | FABP type I-Fh1 | 1 | Fh/Fg | Fh/Fg | Fsp1 |
|  |  |  | FABP type I-Fh20 | 1 |  |  |  |
|  |  |  | FABP type I-Fg36 | 2 |  |  |  |
|  |  |  | FABP type I-Fh1 | 1 | Fh/Fg | Fh/Fg | Fsp1 |
|  |  |  | FABP type I-Fh11 | 1 |  |  |  |
|  |  |  | FABP type I-Fg36 | 1 |  |  |  |
|  |  |  | FABP type I-Fg39 | 1 |  |  |  |
|  |  |  | FABP type I-Fh12 | 1 | Fh/Fg | Fh/Fg | Fsp1 |
|  |  |  | FABP type I-Fh13 | 1 |  |  |  |
|  |  |  | FABP type I-Fg36 | 1 |  |  |  |
|  |  |  | FABP type I-Fg40 | 1 |  |  |  |
|  |  |  | FABP type I-Fh1 | 1 | Fh/Fg | Fh/Fg | Fsp1 |
|  |  |  | FABP type I-Fh18 | 1 |  |  |  |
|  |  |  | FABP type I-Fg1 | 1 |  |  |  |
|  |  |  | FABP type I-Fg36 | 1 |  |  |  |
|  |  |  | FABP type I-Fh11 | 1 | Fh/Fg | Fh/Fg | Fsp1 |
|  |  |  | FABP type I-Fh19 | 1 |  |  |  |
|  |  |  | FABP type I-Fg1 | 2 |  |  |  |
|  |  |  | FABP type I-Fh1 | 1 | Fh/Fg | Fh/Fg | Fsp1 |
|  |  |  | FABP type I-Fh15 | 1 |  |  |  |
|  |  |  | FABP type I-Fg1 | 1 |  |  |  |
|  |  |  | FABP type I-Fg36 | 1 |  |  |  |
|  |  |  | FABP type I-Fh11 | 2 | Fh/Fg | Fh/Fg | Fsp1 |
|  |  |  | FABP type I-Fg1 | 2 |  |  |  |
|  |  |  | FABP type I-Fh1 | 1 | Fh/Fg | Fh/Fg | Fsp1 |
|  |  |  | FABP type I-Fh11 | 1 |  |  |  |
|  |  |  | FABP type I-Fg1 | 2 |  |  |  |
|  |  |  | FABP type I-Fh1 | 2 | Fh/Fg | Fh/Fg | Fh-C4 |
|  |  |  | FABP type I-Fg1 | 2 |  |  |  |
|  | Bangladesh | 10 | FABP type I-Fh1 | 1 | Fh/Fg | Fh/Fg | Fg-NDI-Bd11 |
|  |  |  | FABP type I-Fh16 | 1 |  |  |  |
|  |  |  | FABP type I-Fg35 | 2 |  |  |  |
|  |  |  | FABP type I-Fh1 | 2 | Fh/Fg | Fh/Fg | Fg-NDI-Bd11 |
|  |  |  | FABP type I-Fg35 | 2 |  |  |  |
|  |  |  | FABP type I-Fh1 | 2 | Fh/Fg | Fh/Fg | Fg-NDI-Bd11 |
|  |  |  | FABP type I-Fg35 | 1 |  |  |  |
|  |  |  | FABP type I-Fg44 | 1 |  |  |  |
|  |  |  | FABP type I-Fh1 | 2 | Fh/Fg | Fh/Fg | Fg-NDI-Bd11 |
|  |  |  | FABP type I-Fg35 | 1 |  |  |  |
|  |  |  | FABP type I-Fg42 | 1 |  |  |  |
|  |  |  | FABP type I-Fh1 | 2 | Fh/Fg | Fh/Fg | Fg-NDI-Bd11 |
|  |  |  | FABP type I-Fg35 | 1 |  |  |  |
|  |  |  | FABP type I-Fg41 | 1 |  |  |  |
|  |  |  | FABP type I-Fh1 | 2 | Fh/Fg | Fh/Fg | Fg-NDI-Bd11 |
|  |  |  | FABP type I-Fg37 | 2 |  |  |  |
|  |  |  | FABP type I-Fh1 | 2 | Fh/Fg | Fh/Fg | Fg-NDI-Bd11 |
|  |  |  | FABP type I-Fg35 | 2 |  |  |  |
|  |  |  | FABP type I-Fh1 | 1 | Fh/Fg | Fh/Fg | Fg-NDI-Bd11 |
|  |  |  | FABP type I-Fh17 | 1 |  |  |  |
|  |  |  | FABP type I-Fg35 | 1 |  |  |  |
|  |  |  | FABP type I-Fg43 | 1 |  |  |  |
|  |  |  | FABP type I-Fh1 | 2 | Fh/Fg | Fh/Fg | Fg-NDI-Bd11 |
|  |  |  | FABP type I-Fg37 | 1 |  |  |  |
|  |  |  | FABP type I-Fg38 | 1 |  |  |  |
|  |  |  | FABP type I-Fh1 | 1 | Fh/Fg | Fh/Fg | Fg-NDI-Bd11 |
|  |  |  | FABP type I-Fh14 | 1 |  |  |  |
|  |  |  | FABP type I-Fg35 | 2 |  |  |  |
| total |  | 75 |  |  |  |  |  |

*FABP type I* genotypes obtained in the present study are deposited in the DNA data bank of Japan under accession numbers LC718928–LC718992.

The *nad1* haplotypes were analyzed in the previous studies described in Table 1.

The *nad1* haplotypes from Bangladesh were analyzed in the present study according to Mohanta et al. [19].
